# Inactivity periods and postural change speed can explain atypical postural change patterns of *Caenorhabditis elegans* mutants

**DOI:** 10.1186/s12859-016-1408-8

**Published:** 2017-01-19

**Authors:** Tsukasa Fukunaga, Wataru Iwasaki

**Affiliations:** 1 Department of Computational Biology and Medical Science, Graduate School of Frontier Sciences, The University of Tokyo, Chiba, 277-8568 Japan; 2 Faculty of Science and Engineering, Waseda University, Tokyo, 169-0072 Japan; 3 Research Fellow of Japan Society for the Promotion of Science, Tokyo, Japan; 4 Atmosphere and Ocean Research Institute, The University of Tokyo, Chiba, 277-8564 Japan; 5 Department of Biological Sciences, Graduate School of Science, The University of Tokyo, Tokyo, 113-0032 Japan

**Keywords:** Computational ethology, Eigenworms, Worm posture analysis, Transition patterns of postures

## Abstract

**Background:**

With rapid advances in genome sequencing and editing technologies, systematic and quantitative analysis of animal behavior is expected to be another key to facilitating data-driven behavioral genetics. The nematode *Caenorhabditis elegans* is a model organism in this field. Several video-tracking systems are available for automatically recording behavioral data for the nematode, but computational methods for analyzing these data are still under development.

**Results:**

In this study, we applied the Gaussian mixture model-based binning method to time-series postural data for 322 *C. elegans* strains. We revealed that the occurrence patterns of the postural states and the transition patterns among these states have a relationship as expected, and such a relationship must be taken into account to identify strains with atypical behaviors that are different from those of wild type. Based on this observation, we identified several strains that exhibit atypical transition patterns that cannot be fully explained by their occurrence patterns of postural states. Surprisingly, we found that two simple factors—overall acceleration of postural movement and elimination of inactivity periods—explained the behavioral characteristics of strains with very atypical transition patterns; therefore, computational analysis of animal behavior must be accompanied by evaluation of the effects of these simple factors. Finally, we found that the *npr-1* and *npr-3* mutants have similar behavioral patterns that were not predictable by sequence homology, proving that our data-driven approach can reveal the functions of genes that have not yet been characterized.

**Conclusion:**

We propose that elimination of inactivity periods and overall acceleration of postural change speed can explain behavioral phenotypes of strains with very atypical postural transition patterns. Our methods and results constitute guidelines for effectively finding strains that show “truly” interesting behaviors and systematically uncovering novel gene functions by bioimage-informatic approaches.

**Electronic supplementary material:**

The online version of this article (doi:10.1186/s12859-016-1408-8) contains supplementary material, which is available to authorized users.

## Background

While recent advances in DNA sequencing technology have greatly facilitated genomic analysis, quantitative and reproducible analysis of animal behavior is expected to further promote data-driven behavioral genetics [1–4]. *Caenorhabditis elegans* is a model organism for which various research resources are available, including a high-quality genome sequence, highly curated and integrated databases, and a complete neuronal wiring diagram [5–7]. Several systems that automatically track and video-record individual worms are already available for ethological studies [8–13]. Some of these systems record not only movement trajectories but also time-series postural images of individual worms. Although these trajectories and postures are not independent from each other [14], perturbations at the molecular and cellular levels influence the latter more directly than the former.

Computational methods for analyzing *C. elegans* time-series postural data are still under development. A classic approach is to search given datasets for predefined postural patterns or behavioral parameters; however, such an approach suffers from a lack of objectivity or the ability to identify novel characteristics [15]. A more systematic approach is to use unsupervised machine learning to find frequently appearing stretches or “behavioral motif” *de novo* within time-series postures. Using this approach, Brown et al. analyzed 7708 movies of 307 mutant strains and detected 2223 *C. elegans* behavioral motifs [16]. A feature vector for each individual worm was then calculated based on the detected behavioral motifs, and clustering of the strains using these feature vectors successfully grouped mutant strains in which the responsible genes have related biological functions [16]. Szigeti et al. developed another method for finding behavioral motifs based on spline mixture models and identified motifs corresponding to turning or passive behaviors [17].

An alternative systematic approach for analyzing time-series postural data is to quantify transition frequencies between “postural states”. In this approach, worm postures are clustered based on similarities between postures and the obtained clusters are defined as postural states. Whereas the behavioral motif approach detects atypical behaviors as continuous stretches, the postural state approach detects those that rather reflect worms’ prompt reaction, which might reflect their decision-making criteria, for instance. As a pioneering work, Schwarz et al. used *K*-means clustering to bin worm postures, and observed condition-specific state transition patterns [18]. However, the factors underlying atypical worm postural movement patterns were not sufficiently dissected, particularly because postures and transition patterns between them should not be independent from each other.

Here, we applied the Gaussian Mixture Model (GMM)-based binning method [19] to time-series postural data for 322 *C. elegans* strains to quantify their transition frequencies between postural states, and revealed that the occurrence patterns of the postural states and the transition patterns among these states have a relationship as expected. In addition, we discovered several strains that exhibit atypical transition patterns that cannot be fully explained by their occurrence patterns of postural states. We also propose that elimination of inactivity periods where the postural change speed is nearly equal to zero, and overall acceleration of postural change speed can explain the behavioral phenotypes of strains with very atypical transition patterns.

## Methods

### Dataset preparation

**Fig. 1:**
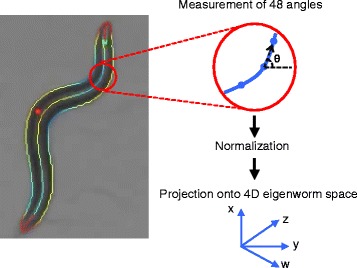
Measurement and eigenworm representation of worm postures. The *left panel* shows a picture of a wild-type N2 worm; its contour and midline are highlighted. This picture was taken from the *C. elegans* behavioral database [20]. In total, 48 angles were measured at regular intervals along the midline and projected onto the four-dimensional eigenworm space after normalization

The original dataset was obtained from the *C. elegans* behavioral database [20] and consisted of data from 9975 hermaphroditic individual worms of 338 strains freely crawling on agar plate surfaces with food. The 338 strains comprised 21 wild-type (including N2) and 317 N2-derived mutant strains. To concisely represent their postures, we adopted four-dimensional eigenworm vectors [20, 21] that were pre-calculated in the original dataset. In brief, an eigenworm vector was calculated from each image frame of a video-recorded individual worm as follows. First, the midline of the worm body was obtained by image processing, and 48 angles were measured at regular intervals along the midline (Fig. 1). Second, the 48 angles were normalized to obtain a mean value of zero to ignore the general orientation of the body. Third, the normalized 48 values were projected onto four dimensions that were defined by four eigenvectors explaining 92% of the overall variability of worm postures [16, 21]. Such an eigenvector representation of animal shapes is widely accepted for analyzing animal behavior [17, 22].

To ensure that the data were consistent, we excluded data from any individual worm whose video length was not between 890 and 910 seconds or whose eigenworm vectors were missing in more than 40% of the entire frames (typically because of video-tracking failure). Missing eigenworm vectors in the remaining dataset were linearly interpolated using values from the two immediately flanking frames (see Additional file 1: Figure S1A for the proportions of such “gap” frames). Because various frame rates were used in the original dataset (Additional file 1: Figure S1B), we downsampled all data to five frames per second. Finally, by excluding data for any strain for which less than five different individuals were available, we obtained time-series eigenworm vector data for 8769 individual worms from 322 strains (20 wild-type and 302 mutant strains).

### Probabilistic binning of *C. elegans* postures into postural states

To represent any eigenworm vector by discrete postural states in a probabilistic manner, we used a GMM-based binning method [19]. This method represents each four-dimensional eigenworm vector by a probabilistic mixture of multiple Gaussian distributions. First, because the total number of image frames in the entire dataset was too large, we randomly sampled 1% of the frames (i.e., 385,790 frames) for parameter estimation. Second, we plotted the eigenworm vectors of all frames and fit the four-dimensional GMM to the pooled distribution consisting of 385,790 data points. The GMM parameters were estimated by the factorized asymptotic Bayes (FAB) algorithm [23]. The FAB algorithm is similar to the conventional expectation-maximization algorithm for fitting GMM [19] but allows automatic estimation of the numbers of mixture components based on factorized information criterion (FIC) [23]. Unlike conventional information criteria such as Akaike information criterion and Bayesian information criterion, FIC can be applied to the inference of mixture models with theoretical justification. The FAB algorithm eliminates components if their mixture ratios are smaller than a given threshold *ε* after the E-step that is modified from that of the conventional expectation-maximization algorithm. The source codes of FAB-GMM algorithm are available at https://github.com/fukunagatsu/FAB-GMM. We set *ε*=0.01,0.005, or 0.001 using initial parameter sets estimated by the *K*-means++ algorithm [24] with *K*=100,200, or 1000, respectively (These *K* values are the maximum numbers of states for each *ε*). Third, after the convergence of the FAB algorithm, each Gaussian distribution component was regarded as a postural state. We obtained 44, 95, and 459 postural states when *ε* was 0.01, 0.005, and 0.001, respectively. Finally, for each frame (including the remaining 99%), the responsibility of each Gaussian distribution component for explaining its eigenworm vector was calculated using the estimated parameters. As a result, a posture of any individual *i* at any frame *f* was represented by a *K*-dimensional nonnegative vector **r**_*i*,*f*_=(*r*_*i*,*f*,1_,*r*_*i*,*f*,2_,…,*r*_*i*,*f*,*K*_)^T^, where *K* is the number of postural states, each element represents the responsibility of each state, and $\sum _{k=1}^{K} r_{{i,f,k}} = 1$.

For comparison, we also adopted the *K*-means clustering, which not probabilistically but deterministically bins any eigenworm vector to a single state. First, the same set of 1% frames were selected and their eigenworm vectors were plotted to the four-dimensional space in the same manner. Second, *K*-means clustering was applied to the pooled distribution. The model parameters were estimated by the Lloyd algorithm [25] with initial parameters estimated by *K*-means++ algorithm [24], where *K* was set to 90 (a parameter used in a previous study [18]) or 44, 95, or 459 (parameters estimated by the GMM-based method earlier). Third, after the convergence of the Lloyd algorithm, a centroid of each cluster was regarded as a postural state. Finally, for each frame (including the remaining 99%), its eigenworm vector was binned to the closest postural state. Note that, any worm posture was represented by a vector **r**_*i*,*f*_ as in the case of the GMM-based method, but it was an integer vector (i.e., only one of its elements was 1 and the others were 0).

### Evaluation of binning methods

In this work, we assumed that the postures of individual worms belonging to the same strain should be statistically more similar than the postures of worms belonging to different strains. Thus, if worm postures are represented more properly by the postural states, the relative state occurrence frequencies of an individual $i, \mathbf {r}_{i} = \frac {1}{F}\sum _{f=1}^{F} \mathbf {r}_{i,f} $, where *F* is the number of frames, are expected to be more similar between individuals of the same strain than those of different strains. The GMM-based method and *K*-means clustering with three and four different parameters, respectively, were compared based on this rationale.

Let *S*_*i*_ be a set of individuals that belong to the same strain as *i* except *i*, and $\overline {S}_{i}$ be a set of randomly selected individuals such that $S_{i} \cap \overline {S}_{i} = \emptyset, i \notin \overline {S}_{i}$, and $|S_{i}| = |\overline {S}_{i}|$, where |*S*| represents the number of individuals belonging to *S*. For every individual *i*, we calculated the mean divergences of the relative state occurrence frequencies against *S*_*i*_ and $\overline {S}_{i} $ as follows:

$$\Delta_{\text{intra}} \mathbf{r}_{i} = \frac{1}{|S_{i}|} \sum_{j \in S_{i}} d(\mathbf{r}_{i}, \mathbf{r}_{j})  $$

$$\Delta_{\text{inter}} \mathbf{r}_{i} = \frac{1}{|\overline{S}_{i}|} \sum_{j \in \overline{S}_{i}} d(\mathbf{r}_{i}, \mathbf{r}_{j})  $$

where *d* is the Jensen-Shannon divergence, which is a measure of divergence between two probability distributions [26]. Note that **r**_*i*_ is a normalized vector and can be regarded as a probability distribution. Then, for each strain, we tested the hypothesis that *Δ*_intra_ **r**_*i*_ of all individuals that belong to that strain are statistically smaller than *Δ*_inter_ **r**_*i*_ of them by a one-sided Wilcoxon-Mann-Whitney test. The test was repeated against all strains, and the Benjamini-Hochberg approach was used to control the false-discovery rates of multiple testing (*q*<0.05) [27].

### Discovery of strains showing wild-type N2-like postures but atypical transition patterns

For each individual *i*, the relative transition frequencies between postural states were represented by a *K*×*K* matrix *T*_*i*_ whose element representing the transition from a state *k* to *l* is

$$T_{\text{\textit{i,k,l}}} = \frac{1}{F-1}\sum_{f=1}^{F-1} r_{\text{\textit{i,f,k}}} r_{i,f+1,l}  $$

For each strain *S*, the relative state occurrence frequency **r**_*i*_ and relative state transition frequency *T*_*i*_ were averaged for its individuals to obtain **r**_*S*_ and *T*_*S*_, respectively. With the exception of the wild-type N2 strain, we calculated the divergences of each strain from wild-type N2 as follows:

$$\Delta_{\text{N2}} \mathbf{r}_{S} = d(\mathbf{r}_{S}, \mathbf{r}_{\text{N2}})  $$

$$\Delta_{\text{N2}} T_{S} = d(T_{S}, T_{\text{N2}})  $$

where *d* is the Jensen-Shannon divergence. Note that *T*_*i*_ can also be regarded as a probability distribution. For example, a large *Δ*_N2_ *T*_*S*_ indicates that strain *S* has a state transition pattern that is very different from that of wild-type N2.

Next, we conducted linear regression to investigate relationships between a dependent variable *Δ*_N2_ *T*_*S*_ and an explanatory variable *Δ*_N2_ **r**_*S*_. Then, we detected strains showing atypical transition patterns using standardized residuals (Z-values) from the estimated linear model. To control the false-discovery rates of multiple testing, we used the Benjamini-Hochberg approach (*q*<0.05).

### Analysis of factors underlying atypical state transition patterns

To reveal factors underlying the atypical transition patterns, we created artificial N2 strains *in silico* by modifying the eigenworm data for the wild-type N2 strain and determined if these artificial N2 strains reproduced the atypical state transition frequencies of strains that showed atypical transition patterns. Specifically, we focused on the effects of eliminating inactivity periods and accelerating the average postural change speed. To remove inactivity periods from wild-type N2, we excluded any frame for which the Euclidean distance of the eigenworm vectors between that and the previous frame was smaller than a threshold *α*. To change the postural change speed as a whole, we removed frames at regular intervals to simulate movement of *β*-times accelerated wild-type N2. When *β*=1.5, for every three consecutive frames, the eigenworm vectors of the second and third frames were replaced with the averaged vector. When *β*=2, every second frame was removed. Because all strains that showed atypical transition patterns were faster than wild-type N2 on average, only acceleration was considered here (i.e., deceleration was not considered here). For each strain *S*, the parameters *α* and *β* were selected to minimize

$$D_{\text{eigenworm speed}} = \int_{x}|F_{\text{aN2}}(x)-F_{S}(x)| dx  $$

where *F*_aN2_ and *F*_*S*_ are the cumulative distributions of the instantaneous postural change speed (Euclidean distance between eigenworm vectors of adjacent frames) of the artificial N2 strain and *S*, respectively.

Then, we calculated **r**_aN2_ and *T*_aN2_, which are the relative state occurrence frequency and relative state transition frequency, respectively, of the artificial N2 strain. To determine whether the artificial N2 strain reproduced the behavioral characteristics of the strain *S*,*Δ*_aN2_ **r**_*S*_ and *Δ*_aN2_ *T*_*S*_. In addition, we calculated the standardized residuals of *Δ*_aN2_ **r**_*S*_ and *Δ*_aN2_ *T*_*S*_ based on the previously predicted linear model. We called this standardized residual *Z*_*a*_.

## Results

### Evaluation of binning methods and parameters

The postures of each of the 8769 individual worms belonging to 322 strains were represented by time-series four-dimensional eigenworm vectors. Every eigenworm vector was binned to postural states by the GMM-based method and *K*-means clustering with three and four parameters, respectively. Then, we determined if the relative state occurrence frequencies were more similar between worms of the same strain than between worms of different strains. The number of strains for which the null hypothesis of no difference with Benjamini-Hochberg’s *q*<0.05 was rejected is shown in Table 1. Overall, the GMM-based method detected greater numbers of such strains than *K*-means clustering, indicating that less postural information was lost during binning in the former than in the latter. Although the parameter selection did not have a strong impact on the results, *K*=95 and *ε*=0.005 were the best parameters for the *K*-means clustering and GMM-based methods, respectively. Among the 213 strains that exhibited significance by *K*-means-clustering with *K*=95, only ten were missed by the GMM-based method with *ε*=0.005 (Additional file 1: Figure S2). Therefore, the GMM-based method with *ε*=0.005 was adopted for postural state binning in the following analyses.

**Table 1:** Evaluation of binning methods and parameters

Binning method	Parameter	Number of strains
*K*-means	*K*=44	207
	*K*=90	203
	*K*=95	213
	*K*=459	205
GMM	*ε*=0.01	238
	*ε*=0.005	**242**
	*ε*=0.001	229

Shown are the numbers of strains whose relative state occurrence frequencies were significantly more similar between those of the same strain than those of different strains (one-sided Wilcoxon-Mann-Whitney test with the Benjamini-Hochberg procedure (*q*<0.05)). The bold values are the highest scores

### Strong relationships between postural state occurrences and transitions

**Fig. 2:**
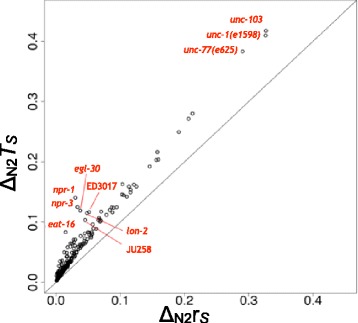
Divergences of postural state occurrence and transition frequencies of 321 non wild-type N2 strains. The *x*- and *y*-axis represent *Δ*_N2_ **r**_*S*_ and *Δ*_N2_ *T*_*S*_, respectively

After the binning of eigenworm vectors to the postural states, the relative state occurrence frequency **r**_*S*_ and relative state transition frequency *T*_*S*_ of each strain *S* were calculated. Figure 2 shows their divergences from those of the wild-type N2 strain, where large *Δ*_N2_ **r**_*S*_ and *Δ*_N2_ *T*_*S*_ indicate that strain *S* displays postures and transition patterns that are very different from those of wild-type N2. We clearly observed a general trend of a positive linear correlation between the two divergence values (The adjusted R-squared value was 0.96). This likely reflected the fact that the use of different postures naturally leads to the use of different transition patterns. Note that *Δ*_N2_ *T*_*S*_−*Δ*_N2_ **r**_*S*_≥0 (the proof is provided in the Additional file 1).

**Fig. 3:**
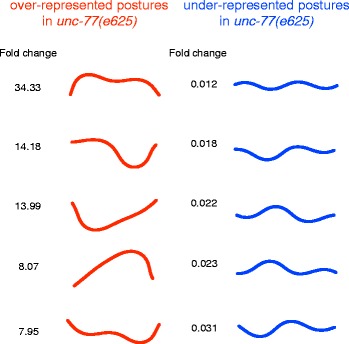
Top five over-represented and under-represented postures in *unc-77(e625)* Each posture was reconstructed from the mean value of the corresponding postural states. *Red* and *blue* colors represent over-represented and under-represented postures in *unc-77(e625)*, respectively

For example, three mutant strains, *unc-103*, *unc-1(e1598)*, and *unc-77(e625)*, exhibited the largest divergences of both values from wild-type N2 (Fig. 2). The *unc-103* gene encodes an ether-a-go-go-related *K* ^+^ channel homolog, and the strain in which this gene has a gain-of-function mutation has been reported to show extremely lethargic behavior [28]. The *unc-1(e1598)* strain is a mutant of a stomatin-like-protein gene and has also been reported to show very slow behavior [29]. The large deviations of the postural state occurrence and transition frequencies of these two mutant strains likely reflect their exceptionally inactive phenotypes. The *unc-77(e625)* strain features a gain-of-function mutation of a subunit gene of a voltage-insensitive cation leak channel and exhibits coiled postures [29]. To reveal what postures are specific in this strain, we calculated the fold change between **r**_*unc−77(e625),k*_ and **r**_N2,*k*_ for each postural state *k* and detected over-represented and under-represented postures in *unc-77(e625)* (Fig. 3). These results showed that the *unc-77(e625)* strain tends to take more “C-shaped” but less “S-shaped” postures compared to the wild-type N2 strain.

### Identification of strains exhibiting atypical transition patterns

**Fig. 4:**
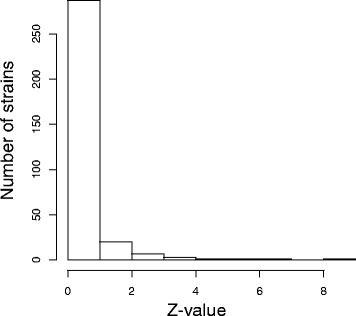
Histogram of *Z*-values of 321 non wild-type N2 strains

As shown in Fig. 2, although most strains strongly followed the positive linear correlation trend, several strains did not. We identified seven strains exhibiting atypical transition patterns that were significantly deviated from expectation (*q*<0.05, left side of Fig. 2). Only these seven strains showed Z-values larger than 3.0 (Table 2, Fig. 4).

**Table 2:** Strains exhibiting wild-type N2-like postures but atypical transition patterns

Strain	*Δ*_N2_ **r**_*S*_	*Δ*_N2_ *T*_*S*_	Z-value
*npr-1*	0.0299	0.1404	8.192
*npr-3*	0.0333	0.1249	6.423
*egl-30*	0.0376	0.1192	5.436
*eat-16*	0.0149	0.0831	4.872
*lon-2*	0.0481	0.1150	3.876
ED3017	0.0514	0.1167	3.632
JU258	0.0453	0.1033	3.163

The two strains with the largest Z-values, *npr-1* and *npr-3*, are mutants of neuropeptide receptor (*npr*) genes. As it is known that NPR-1 is expressed in ventral nerve cord motor neuron, it is reasonable that these neuropeptide receptor genes have roles in controlling postural movement [30, 31]. However, notably, other *npr* mutant strains did not show large Z-values, even though *npr-1* and *npr-3* are neither the closest paralogs in the *npr* gene family nor highly identical in sequence (amino-acid sequence identity% = 25.5) (Additional file 1: Figure S3). Because **r**_*S*_ and *T*_*S*_ of these two strains were most similar to each other among all strains (i.e., the differences of **r**_*S*_ and *T*_*S*_ between the two strains were the smallest among every strain pair that contains either of the *npr-1* and *npr-3* strains), the *npr-1* and *npr-3* genes were suggested to have closely related functions at the behavioral level regardless of their different evolutionary origins at the sequence level.

The *egl-30* and *eat-16* genes encode components of heterotrimeric G-protein signaling pathways. Loss of EGL-30 function depresses the behavioral activity of *C. elegans*, whereas EAT-16 negatively regulates EGL-30 [32, 33]. Because the *egl-30* and *eat-16* mutant strains in this study have gain- (*ep271*) and loss-of-function alleles (*sa609*), respectively [33, 34], their similar, active behavioral phenotypes are consistent with previous reports. Indeed, **r**_*S*_ and *T*_*S*_ from the *egl-30* and *eat-16* strains were most similar to each other.

*lon-2* encodes a glypican-family protein of heparan sulfate proteoglycans, and its mutant was reported to have a longer body than that of wild-type N2 [35]. A previous study reported that *lon-2* was one of the worst-fit mutants in the eigenworm representation [16]. Although it is not clear why *Δ*_N2_ **r**_*S*_ of *lon-2* is not very large, the poor fitting of the eigenworm representation may have resulted in atypical transition patterns of this strain.

ED3017 and JU258 are non-N2 wild-type strains. *C. elegans* population genomics studies revealed that N2 strains acquired gain-of-function mutations in *npr-1* during laboratory domestication [36, 37], and ED3017 and JU258 have a lower activity allele in *npr-1*. The large Z-values of these two strains may be caused by this low *npr-1* activity.

### Analysis of factors underlying atypical state transition patterns

**Fig. 5:**
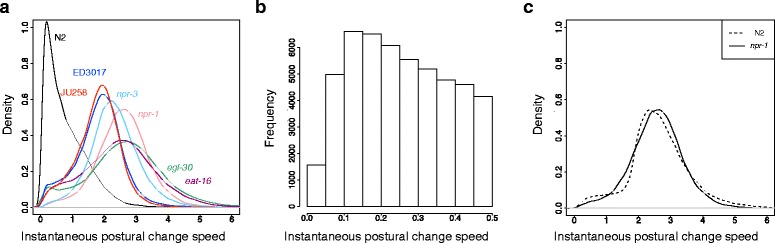
Distributions of instantaneous speed of postural change. **a** The distributions of wild-type N2, *npr-1*, *npr-3*, *egl-30*, *eat-16*, ED3017, and JU258. The *y*-axis represents density. **b** A histogram that magnifies around the mode of the wild-type N2 distribution. **c** The distributions of *npr-1* and the artificial N2 strain whose postural change speed resembles that of *npr-1*

Figure 5 a presents the distributions of the instantaneous postural change speed of wild-type N2 and the six strains that exhibited atypical transition patterns. Note that *lon-2* was excluded here because the earlier stage of eigenworm representation could be problematic for this strain. Overall, all six strains exhibited faster postural change speeds than those of wild-type N2. Only wild-type N2 had a mode of the postural change speed at approximately 0.1 (Fig. 5 b), at which four of the other strains also had small “shoulders” (Fig. 5 a). Such a small speed value indicates that the individuals are under inactivity periods, which may correspond to *quiescence* worm behavior [38]. We also observed several strains that retain this mode of postural change speed at approximately 0.1 but have different distribution shapes from that of wild-type N2 (e.g., *unc-43* and *C52B9.11*; Additional file 1: Figure S4).

On the basis of these observations, we investigated whether artificial elimination of the inactivity periods and overall acceleration of postural change speed from wild-type N2 could reproduce the state transition patterns of these six strains without significantly altering the state occurrence frequencies. In time-series sequence representations of postural states, inactivity periods are represented by stretches of identical or similar state(s). Because inactivity periods likely do not occur only at specific postural states (the Jensen-Shannon divergence of relative state occurrence frequencies between frames whose posture change speed was less than and greater than 0.3 was 0.0161 for wild-type N2), the elimination of inactivity periods would change the state transition frequencies while modestly preserving the state occurrence frequencies. For the acceleration of postural change speed, for example, two-fold acceleration of a state sequence AABBCCAA... into ABCA... as a whole will also change the state transition frequencies by preserving the state occurrence frequencies.

Artificial N2 strain data were created *in silico* by removing inactivity periods with threshold *α* and accelerating *β*-fold as a whole from the time-series eigenworm data for wild-type N2. For each of the six strains, we chose the best parameters from *α*=0.3,0.4,…,1.0 and *β*=1.5,2.0 by examining whether the postural change speed distributions of the artificial N2 strains fit those of each of the six strains (Fig. 5 c, Additional file 1: Figure S5, and Additional file 1: Table S1). Finally, we investigated whether the artificial N2 strains reproduced not only the distributions of the instantaneous postural change speed but also the postural state occurrence and transition frequencies of the six strains. All *Z*_*a*_ values became substantially smaller than the original Z-values, and these values decreased to the level that was not significantly different from expectation (Table 3). In other words, the atypical state transition frequencies of these four mutant strains can be explained almost entirely by the lack of inactivity periods and overall acceleration of the postural change speed.

**Table 3:** Reproduction of atypical state transition frequencies by artificially modified N2

Strain	*Δ*_aN2_ **r**_*S*_	*Δ*_aN2_ *T*_*S*_	*Z*_*a*_	Original Z-value
*npr-1*	0.0135	0.0314	0.484	8.192
*npr-3*	0.0180	0.0426	0.953	6.423
*egl-30*	0.0235	0.0494	0.921	5.436
*eat-16*	0.0147	0.0307	0.285	4.872
ED3017	0.0351	0.0695	1.360	3.632
JU258	0.0290	0.0577	1.021	3.163

## Discussion

In this study, we used the GMM-based method for probabilistically binning worm postures into a finite number of postural states and revealed an apparent relationship between the postures and transition patterns of *C. elegans* strains. The superior binning performance of the GMM-based method reflects the fact that time-series postures of any individual are distributed along a single trajectory in the four-dimensional eigenworm space because the postures of continuous frames should be similar to each other. Thus, a worm must adopt intermediate postures while changing its posture from one postural state to another. Deterministic binning of such intermediate postures inevitably loses information or introduces noise to the representation of worm behavior. The case of the *lon-2* strain in this study also indicates the importance of preserving information during the computational analysis of animal behavior, although the difficulty in this case occurred during the eigenworm representation. The strong relationship between the postural state occurrence and transition frequencies offers two important suggestions for worm postural movement analysis: a significant part of postural movement variations can be evaluated solely by examining postures without temporal information, and the effects of using different postures must be taken into account in postural movement analysis.

Several strains that exhibited atypical transition patterns among postural states were identified. Surprisingly, for the six strains that showed the most atypical postural movement, merely eliminating the inactivity periods and accelerating the postural change speed as a whole nearly reproduced their atypical transition patterns. While quantification of the transition frequencies between postural states is a powerful approach for computationally analyzing animal behavior, our results demonstrate that even very atypical state transition patterns can result from simple factors. Analyses of inactivity periods and postural change speeds both require consideration of time duration; the compression of state time duration abolishes their effects in the analysis [18]. To effectively detect strains that show truly interesting behavior, e.g., strains whose neural circuits encode special decision-making criteria, computational analyses of animal behavior must be accompanied by evaluation of the effects of more “trivial” factors such as overall change in speed (of course, we note that these trivial factors themselves would also provide many insights into worm behavior). The *C. elegans* behavioral database also contains various behavioral data such as dorsal/ventral orientations, velocities, and trajectories during worm movement. Using these additional datasets, we may dissect factors that underlie interesting phenotypes more deeply, for example, effects of dorsal/ventral biases in postural change patterns and/or relationships between postural change patterns and movement trajectories.

Our analysis also revealed that the *npr-1* and *npr-3* genes have closely related functions that were unpredictable by sequence homology, the most basic principle in this genomic era. Many studies have conducted functional analyses of *npr-1* [31, 39–41], but few studies have focused on *npr-3* [42]. Therefore, we envision that existing knowledge about *npr-1* will substantially accelerate future functional analyses of *npr-3* based on the present result.

In this study, divergence of the state occurrence and transition frequencies from the wild-type N2 strain was examined. Although this would make sense for the analysis of N2-derived mutant strains, comparison among different wild-type strains can also be done by selecting another strain as a reference. We expect that the linear correlation trend between the state occurrence and transition frequencies will be recovered regardless of the reference strain choice; however, for example, it would also be of interest to select strains that have specific evolutionary context or strains that show characteristic behavior (such as ED3017 or JU258).

## Conclusions

Although more than a decade has passed since the genomes of many model organisms were sequenced, significant numbers of genes remain functionally uncharacterized. Systematically deciphering their functions beyond straightforward sequence homology analysis is one of the most important goals in computational biology today, where an advantage of bioimage informatics for functional analysis is the ability of this method to directly evaluate phenotypes [43–45]. Finally, it should be noted that genome-editing technologies are enabling rapid construction of genetically engineered animal strains [46]. Bioimage-informatic analysis of their behaviors will, for example, contribute to the identification of novel genes responsible for neurological disease. We emphasize that further development of computational methods and accumulation of technical knowledge will be critical to promote this emerging field.

## Additional file

Additional file 1 Supplementary materials. This file includes additional texts, figures and tables not shown in the manuscript. (PDF 477 kb)

## Abbreviations

FAB: Factorized asymptotic Bayes
FIC: Factorized information criteria
GMM: Gaussian mixture model
