# Supplementary material for: Inactivity periods and postural change speed can explain atypical postural change patterns of Caenorhabditis elegans mutants
Source: BMC Bioinformatics. 2017 Jan 19;18:46. doi: 10.1186/s12859-016-1408-8 (PMC5244558; doi:10.1186/s12859-016-1408-8)
Supplement: Supplementary file 1 — Supplementary materials. This file includes additional texts, figures and tables not shown in the manuscript. (PDF 477 kb) [file 12859_2016_1408_MOESM1_ESM.pdf]

Supplementary Materials for Inactivity periods and postural change speeds can explain atypical postural change patterns of *Caenorhabditis elegans* mutants.

Tsukasa Fukunaga and Wataru Iwasaki

Supplementary Text

Proof of $\Delta_{\text{N2}} T(S) - \Delta_{\text{N2}} \boldsymbol{r}(S) \geq 0$

According to the definition, $\sum_{k=1}^{K} r_k(S) = 1$ and $\sum_{k=1}^{K} \sum_{l=1}^{K} T_{k,l}(S) = 1$. If the videos are of sufficient length, we can ignore the state occurrence frequencies at the last (F-th) frame and obtain $\sum_{l=1}^{K} T_{k,l}(S) = r_k(S)$. In addition, using the GMM-based method, we can assume $0 < r_k(S) < 1$ and $0 < T_{k,l}(S) < 1$ because any postural state can explain any eigenworm vector with a positive probability. By letting $T(S') = (T(S) + T(\text{N2}))/2$ and $\boldsymbol{r}(S') = (\boldsymbol{r}(S) + \boldsymbol{r}(\text{N2}))/2$, the equations and inequalities above also hold true for S'. By representing the Kullback-Leibler divergence by d_{KL}, we obtain

$$\begin{aligned}
\Delta_{\text{N2}} T(S) - \Delta_{\text{N2}} \boldsymbol{r}(S) &= d(T(S), T(\text{N2})) - d(\boldsymbol{r}(S), \boldsymbol{r}(\text{N2})) \\
&= \frac{1}{2} \left(d_{\text{KL}}(T(S), T(S')) - d_{\text{KL}}(\boldsymbol{r}(S), \boldsymbol{r}(S') \right) \\
&\quad + \frac{1}{2} \left(d_{\text{KL}}(T(\text{N2}), T(S')) - d_{\text{KL}}(\boldsymbol{r}(\text{N2}), \boldsymbol{r}(S')) \right)
\end{aligned}$$

Proofs that demonstrate that the first and second terms of the right side are greater than or equal to zero will be given in the same manner. As a representative example, the first term is expanded as follows:

$$\begin{aligned}
& d_{\text{KL}}(T(S), T(S')) - d_{\text{KL}}(\boldsymbol{r}(S), \boldsymbol{r}(S')) \\
&= \sum_{k=1}^{K} \sum_{l=1}^{K} T_{k,l}(S) \log \frac{T_{k,l}(S)}{T_{k,l}(S')} - \sum_{k=1}^{K} r_k(S) \log \frac{r_k(S)}{r_k(S')} \\
&= \sum_{k=1}^{K} \sum_{l=1}^{K} T_{k,l}(S) \log \frac{T_{k,l}(S)}{T_{k,l}(S') r_k(S) / r_k(S')}
\end{aligned}$$

From Jensen's inequality under the condition that $0 < T_{k,l}(S)$ and $0 < T_{k,l}(S')r_k(S)/r_k(S')$, we obtain

$$\begin{aligned}
&\geq -\log \sum_{k=1}^{K} \sum_{l=1}^{K} \frac{T_{k,l}(S')r_k(S)}{r_k(S')} \\
&= -\log \sum_{k=1}^{K} r_k(S) \\
&= 0
\end{aligned}$$

Supplementary Figures

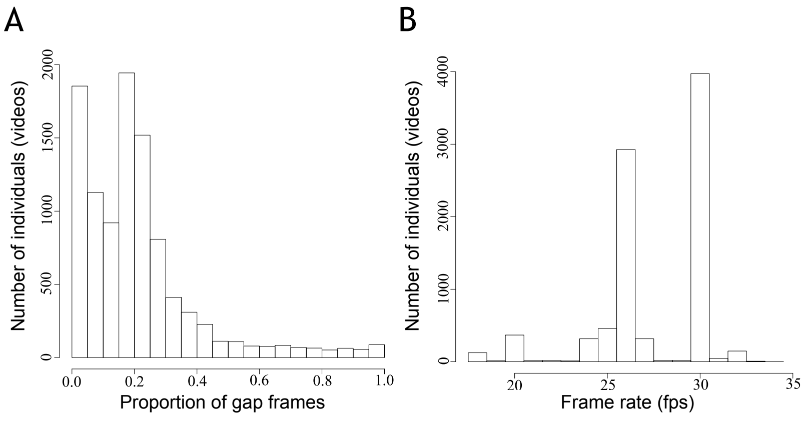

Fig. S1 Number of individuals (i.e., videos) according to (A) proportion of "gap" frames whose eigenworm vectors were missing and (B) frame rates.

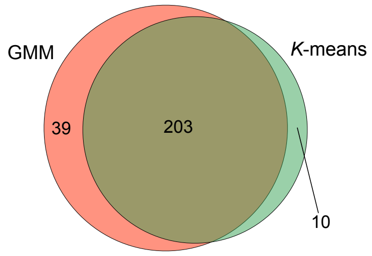

Fig. S2 A Venn diagram of strains whose relative state occurrence frequencies were significantly similar in intra-strain comparison than in inter-strain comparison. The binning methods were the GMM-based method ($\epsilon = 0.005$) and K-means clustering ($K = 95$).

A

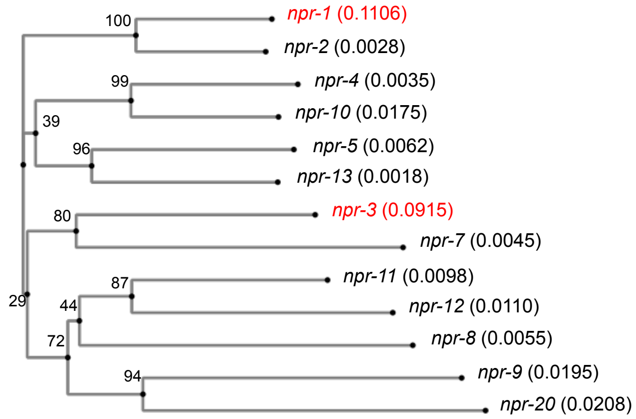

B

```
npr-1           MEVENFTDCQV---YWKVYPDPSQSIYAIVPFLTVYLFLFFLGLFGNVTLIYVTCSHKAL
npr-3           M--EGGRNCVMTVQQWQPEYNDMNQIRAIFSLL--YLLVWVGAIVGNTLVLYVLTFNQVS
                *  *.  :* :    *:   :  :.* **..:*  **:::. .:.**. ::**   ::.

npr-1           LSVQNIFILNLAASDCMMCILSLPITPITNVYKNWYFGNLLCHLIPCIQGISIFVCTFSL
npr-3           LSVRTVFVGCLAGSDLLMCLFSLPITAISIFSRVWVFPAIFCKLIGVFQGGTIFVSSFTL
                ***:.:*:  **.** :**::*****.*: . : * *  ::*:**  :** :***.:*:*

npr-1           GAIALDRYILVVRPHSTPLSQRGAFLTTVLLWILSFVVTLPYAFNMQMIEYTEERICGYF
npr-3           TVIALDRCVLILRPNQEIVNFPRAVFIVFCIWLLGYSLALPVGIYSDIAVYDE--ICGTF
                 .***** :*::**:.  :.   *.: .. :*:*.: ::** .:  ::  * *  *** *

npr-1           CTEKW-----ESAKS--RRAYTMIVMLAQFVVPFAVMAFCYANIVSVLSKRAQTKIRKMV
npr-3           CEENWPDFNPDTGRSGIRRAYGLSVLVLQFGIPALISSICYWMISRVMSDQLARRRGHNI
                * *:*     ::.:*  **** : *:: ** :*  : ::**  *  *:*.:   :  : :

npr-1           ERTSALESSCAFPSHGLEQYENELNEFLDKQEKEKQRVVLQNRRTTSILVTMVVWFGITW
npr-3           RPES-----------------------------ETKLVNRKTRANRMMIVMVVGFVLAW
                .  *                             : ::* :: *:. :::.*** * ::*

npr-1           LPHNVISLIIEYDDTQSFFRLYGRDDYDISYLLNLFTHSIAMSNNVLNPVLYAWLNPSFR
npr-3           MPFNAVNL---YRDLFGISKWYS--------TVFALCHVCAMCSAVLNPIIYSWFNPQFR
                :*.*.:.*   * *  .: : *.         :  : *  **.. ****::*:*:**.**

npr-1           QLVIKTYFGDRRKSDRIINQTSVYKTKIVHDTKHLNGRAKIGGGGSHEALKERELNSCSE
npr-3           QSITTLFKGTDEA--RLIKKKPQSTSKMVSYPTNFS-----------EIRKETEIASTKT
                * : . : *  .   *:*::..  .:*:*  ..::.           *  ** *: * .

npr-1           NLSYHVNGHTRTPTPEVQLNEVSSPEISKLVAEPEELIEFSVNDTLV
npr-3           KITIAENDY------------------------------RAGDQLL
                :::   *.:                              ..* *:
```

Fig. S3 (A) $\Delta\Delta_{\mathrm{N2}}T(S)$ of the *npr*-gene mutants (in parentheses) laid onto an unrooted phylogenetic tree of the *npr* genes. The amino-acid sequences were aligned using MAFFT version 7.271 [1] with the default options and the neighbor-joining phylogenetic tree [2] was reconstructed using the WAG model [3] and all gap-free sites. (B) The amino-acid alignment of the *npr-1* and *npr-3* genes.

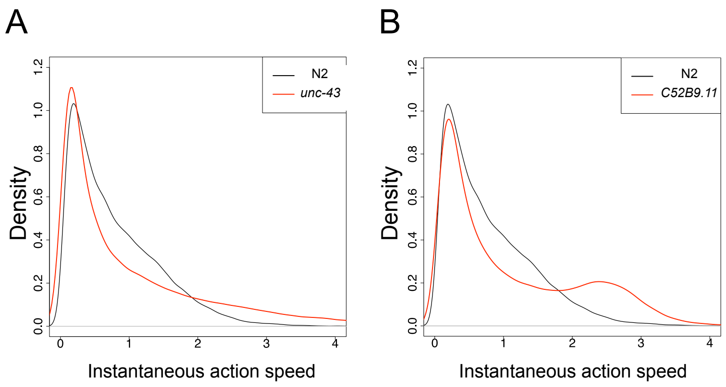

Fig. S4 Distributions of instantaneous speed of postural changes of (A) *unc-43* and (B) *C52B9.11*. The distribution of wild-type N2 is overlaid for comparison.

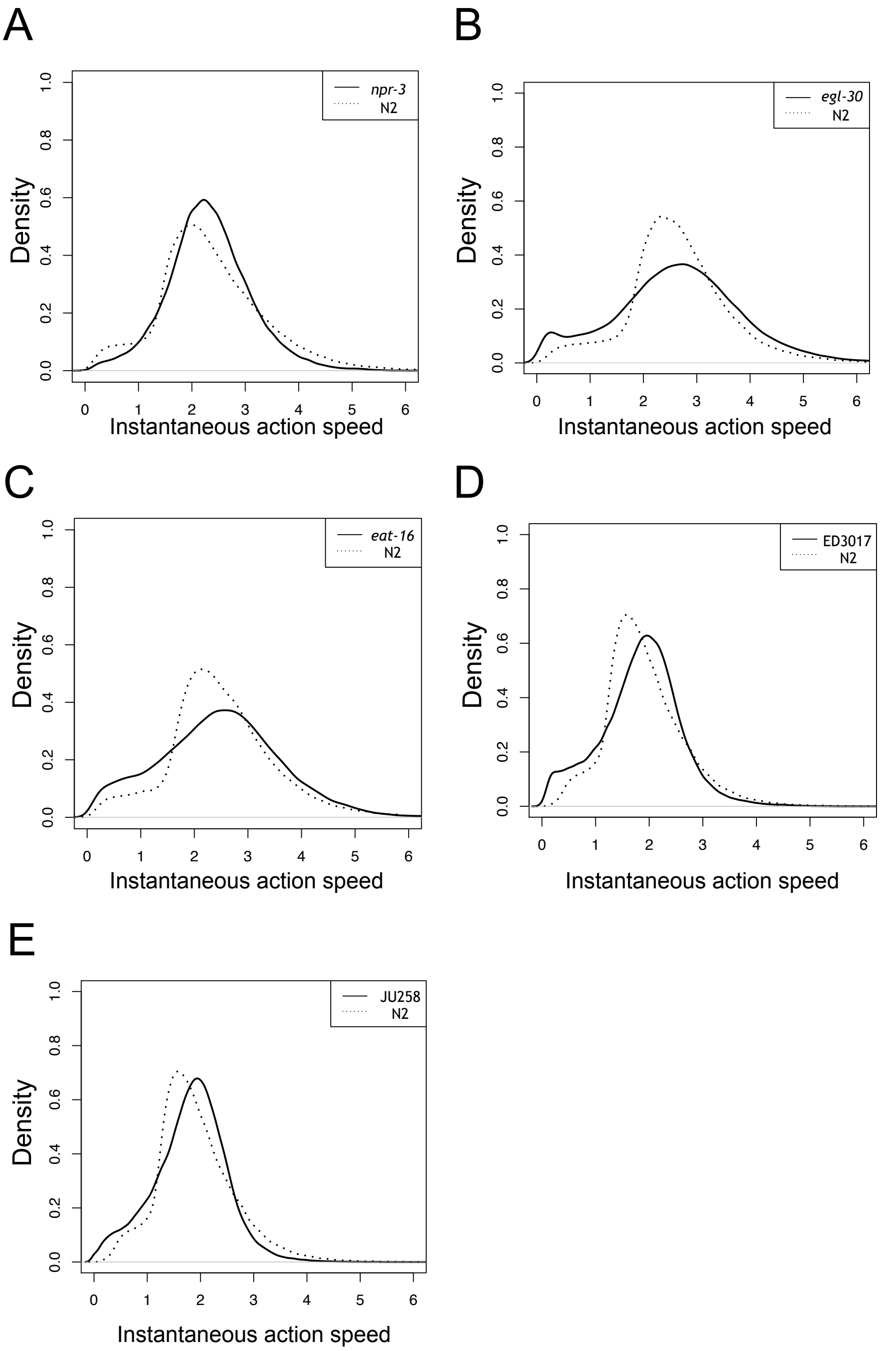

Fig. S5 Distributions of instantaneous speed of postural change of artificial N2 strains that were modified to fit with those of (A) *npr-3*, (B) *egl-30*, (C) *eat-16*, (D) ED3017, and (E) JU258.

Supplementary Table

Table S1 $D_{\text{eigenworm speed}}$ between the artificial N2 and six strains that show similar postures but different transition patterns from wild-type N2, with different parameters α and β.

Strain	β	$\alpha = 0.3$	0.4	0.5	0.6	0.7	0.8	0.9	1.0
npr-1	2	0.802	0.680	0.543	0.416	0.288	0.173	**0.101**	0.246
	1.5	1.213	1.100	0.995	0.882	0.781	0.665	0.563	0.438
npr-3	2	0.583	0.474	0.354	0.247	**0.162**	0.214	0.339	0.494
	1.5	0.954	0.841	0.736	0.624	0.523	0.407	0.305	0.182
egl-30	2	0.830	0.700	0.564	0.451	0.349	0.267	**0.241**	0.262
	1.5	1.270	1.159	1.106	0.960	0.872	0.783	0.707	0.626
eat-16	2	0.671	0.540	0.397	0.295	0.216	**0.175**	0.214	0.335
	1.5	1.108	0.996	0.893	0.789	0.706	0.623	0.557	0.492
ED3017	2	0.361	0.297	0.271	0.380	0.521	0.670	0.808	0.970
	1.5	0.481	0.375	0.280	0.204	0.146	**0.121**	0.179	0.305
JU258	2	0.397	0.336	0.300	0.414	0.554	0.703	0.842	1.003
	1.5	0.473	0.372	0.276	0.197	0.139	**0.127**	0.212	0.338

Reference

[1] Katoh, Kazutaka, and Daron M. Standley. "MAFFT multiple sequence alignment software version 7: improvements in performance and usability." Molecular biology and evolution 30.4 (2013): 772-780.

[2] Saitou, Naruya, and Masatoshi Nei. "The neighbor-joining method: a new method for reconstructing phylogenetic trees." Molecular biology and evolution 4.4 (1987): 406-425.

[3] Whelan, Simon, and Nick Goldman. "A general empirical model of protein evolution derived from multiple protein families using a maximum-likelihood approach." Molecular biology and evolution 18.5 (2001): 691-699.
